# Evidence from multifeature whole-report in visual short-term memory suggests that not all misbinding is swapping

**DOI:** 10.1038/s41598-026-52649-7

**Published:** 2026-05-23

**Authors:** Younes Adam Tabi, Masud Husain, Sanjay Manohar

**Affiliations:** 1 Department of Neurology, University Medical Centre Kiel, Kiel, Germany; 2 Nuffield Department of Clinical Neurosciences, University of Oxford, Oxford, UK; 3 Department of Experimental Psychology, University of Oxford, Oxford, UK

**Keywords:** Mathematics and computing, Neuroscience, Psychology, Psychology

## Abstract

Forgetting is an everyday part of life but its precise mechanisms are incompletely understood. Recall errors are often not random. Rather, people often incorrectly report information about the wrong object in memory. In short term memory, this has been referred to as “misbinding”. Here, it has commonly been assumed that the features of an object get *swapped around* in mind. However, an alternative mechanism is that information about a feature of one object might be lost, and replaced by another object’s feature. Commonly-used cued recall approaches are blind to this distinction, but testing multiple objects on the same trial has the power to detect this. We asked people to report all features of all objects from an array (*multifeature whole-report*) in any order (*free recall*). This enabled us to directly quantify these subtypes of misbinding in memory. We introduce a probabilistic model that shows that misbinding actually includes a mixture of symmetric swaps and *asymmetric* misattributions where a forgotten feature gets replaced by a feature of another object in memory without a reciprocal exchange. This distinction is observed even when memory objects are encoded sequentially.

**Supplementary Information:**

The online version contains supplementary material available at 10.1038/s41598-026-52649-7.

## Introduction

A central feature of memory is the ability to bind together different kinds of information into objects. When we forget, we might forget individual features, whole objects, or how the set of features map to objects – i.e. how they are bound. When people incorrectly report the feature of a different object, this is sometimes termed ‘misbinding’.

**Fig. 1:**
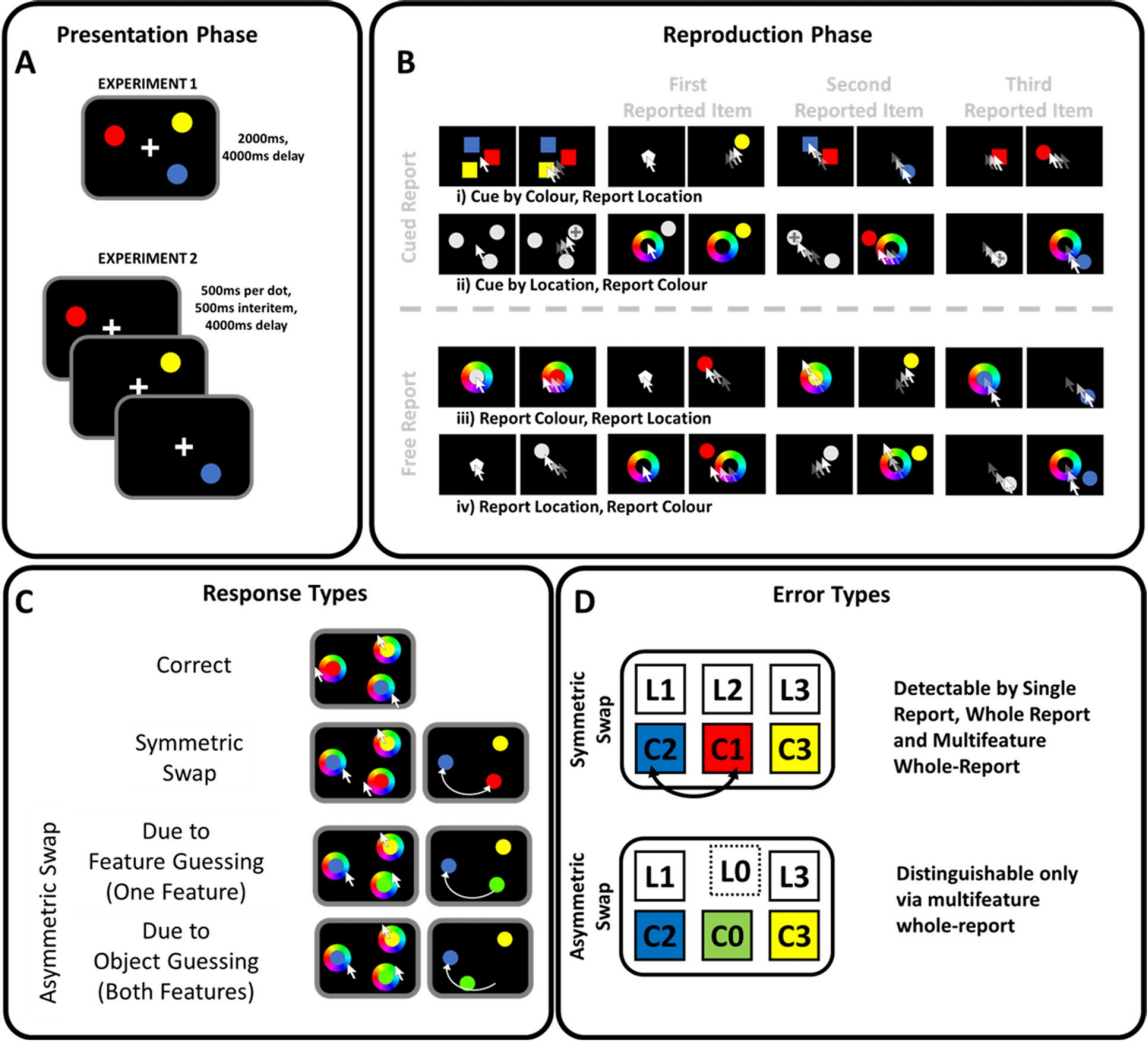
Design of Experiments 1 and 2. In Experiment 1, participants were presented with three coloured dots simultaneously and in Experiment 2 sequentially **(Panel A**,** Presentation Phase)**. After a delay of 4000 ms, participants reported all three objects, on both dimensions, in a freely-chosen order. They were either presented with a cue (**Panel B**,** Reproduction Phase**: top half, “Cued Report”) or were to reproduce the array without a cue (Reproduction Phase: bottom half, “Free Report”). In case of cued recall, participants were either presented with three squares in the colour of the previously seen stimuli **(i)** or with three dots in the locations of the originally presented array **(ii)**. In the free report trials, they either started off with a colour wheel **(iii)** and coloured a dot in the centre of the screen or they placed a grey dot on the boundaries of the circle **(iv)**. Afterwards, they completed the response on the other dimension. **Panel D** named **Response types** depicts the correct order of reported features at the top (locations one to three (L1-3) and colours one to three (C1-3) were to be reported by the participant). In symmetric misbinding, two features on one dimension, colour in this example, are exchanged for one another **(Error Types)**. In symmetric misbinding errors, information on one dimension is bidirectionally exchanged between two objects. However, if there is feature or object guess involved in one of the features, the error becomes an asymmetric misattribution. In feature guessing, participants report a random feature (in this example the colour green OR the location LX) instead of the original feature. In object guessing, both features of an object are forgotten by the participant (colour green AND location LX).

One way binding is commonly tested is by *cued recall*: where participants are asked to remember one feature of an object given another cue feature^1^. “Misbinding errors” can be isolated by modelling errors as coming from one of three possible sources. The information could originate from (i) a distribution centred around the target, (ii) around a non-target (misbinding) or, (iii) a uniform distribution in the case where participants forgot the features altogether^1,2^. This measure of misbinding errors indexes whether a feature from one object is incorrectly bound to a different object. For example, if participants encoded three dots coloured red, yellow and blue, and were later asked to report the colour at a given location (Fig. 1, **Presentation Phase** *and* **Reproduction Phase**), they might report the colour blue instead of red (Fig. 1, **Response Types** *and* **Error Types**).

However, reporting a single feature does not reveal the true fate of the unprobed objects, or importantly, the unprobed features of the probed object. In the above example, participants might report the blue dot’s colour for the originally red dot, but it remains unknown if they would also report the red colour if they were instead probed at the location of the blue dot (Fig. 1, **Response Types**). If they did, we could call this a *symmetric* swap in which two features on one dimension are actually exchanged for one another. This contrasts with *asymmetric* (unidirectional) misattribution, when the blue colour is reported for the originally red dot, but the originally blue dot is not reported reciprocally as being red.

One way to distinguish these two kinds of error (symmetric misbinding vs. asymmetric misattribution) is to use “whole report”, where participants remember a set of objects, and after a short delay, report a feature of *each* of the objects they had previously seen^3–5^. Symmetric misbinding would result in two errors on the same trial. However, correlated forgetting of two objects might arise simply because of poor attention on some trials during encoding. Furthermore, cued and whole report measures may not be comparable, since recall order is not matched. To control for this would require freely-ordered responses even for cued recall.

To summarise, most existing tasks cannot address the critical question of whether misbinding errors are really symmetric. If a feature to be reported was forgotten or misattributed to another object, these tasks do not provide data on what happened to the other features on the *cue* dimension. Specifically, participants might report swapped features merely because they *forgot* the cue features. We suggest this scenario should not be considered a symmetric swap.

Recent evidence from confidence judgements, suggests that we need to distinguish between different kinds of misattribution errors. In those studies, participants recall a feature, and then report their confidence in the response. Non-target responses are most common in low confidence trials compared to high confidence trials^6^, hinting that misbinding is in fact *not* a highly confident response due to a supposed exchange of feature representations. Pratte,^7^ argues that some non-target responses might in fact be strategic guesses. He distinguishes two different types of non-target responses, made with high versus low confidence. In fact, only low confidence non-target responses were evenly distributed relative to the probed object, suggesting they were in fact guesses. High confidence non-target responses, in contrast, were reported near the non-target that was closest to the target on the cue dimension, in line with interference models of retrieval^8^. Hence, cue forgetting can in fact cover up guessing, if only one dimension is reported at a time.

One way to address the question of misattribution is to ask participants to report two features of the *same* object, which we will term *multifeatured whole-report*. In this situation, forgetting one dimension of an object is only weakly coupled to forgetting of the other dimension, suggesting that forgetting afflicts individual features more often than whole objects^2^. However, this would still be in keeping with the existence of either symmetric misbinding or asymmetric misattribution, and so does it not distinguish these scenarios.

Here in two experiments, we introduce a novel method of *multifeature whole-report*. This design allows participants to reproduce *all* features of *all* objects, in the absence of a cue. Together with a new statistical model of sources of error, this enables us to dissociate symmetric misbinding errors from asymmetric misattributions, and feature guessing from object guessing in short-term memory.

Our design requires participants to report both the colour and location of a stimulus. Previous work has suggested that binding to spatial location may be privileged^9–11^. Thus, we counterbalanced the order in which the features had to be recalled. Equally important to spatial binding, may be binding in time. If stimuli are presented simultaneously, competition between features could be increased and symmetric swaps might occur more frequently. The sequential presentation of objects could, on the other hand, increase attentional demand at encoding, increasing interference between objects. Previous studies have found higher error either in sequential^12,13^ or in simultaneously presented arrays^14,15^ in comparison to the other, and so we ran both simultaneous and sequential versions of the experiment.

The nature of objects in memory has been described in various ways, such as ‘files’, ‘pointers’ or ‘slots’, implying mechanisms of keeping features together. More recently, neural and computational accounts of this have been proposed^31,32^ which make predictions about how combinations of features may be associated together. Crucially, the present study challenges the predictions made by such auto-associative memory models, which rely on cue-driven mechanisms to explain retrieval. Auto-associative models typically involve a network of interconnected neurons that facilitate recall by reconstructing a memory trace from partial or degraded inputs (i.e.,^16,17^. However, in the context of multifeature whole-report recall, where no cues are provided, these models may fall short in explaining how individuals retrieve specific details from memory.

Moreover, commonly used mixture models of response error^18,19^ might in fact underestimate guessing for misbinding errors in cued recall experiments. I.e., should an asymmetric misattribution occur, one of these models might in fact predict a classical symmetric misbinding instead of a partial misattribution paired with guessing of one feature.

In summary, we hypothesise that there are asymmetric misattributions as well as symmetric misbindings. We furthermore aim to explore differences in these swap types between simultaneously and sequentially presented objects, and when participants must first reproduce the location or the colour of an object.

## Methods

### Participants

For experiments 1–2, two different groups of 20 participants were recruited. Inclusion criteria were the age range of 18 to 30 and being neurologically normal. Participants were invited for two one-hour sessions that were to take place within a period of seven days. In experiment 2, the 20th participant was not able to perform their second session due to a general lockdown imposed by the British government over the global health incident related to COVID-19. Their data was therefore excluded for analysis.

The study and all its experimental protocols were approved by the local ethics committee of the University of Oxford in accordance with relevant guidelines and regulations. Participants gave their informed consent.

### Stimuli

Participants were presented with stimuli at a viewing distance of approximately 60 cm on a 24-inch LCD monitor. Three dots (radius of 0.7° of visual angle) on a black background were presented in three randomly chosen locations along the boundary of an imaginary circle with a radius of 6.5° around fixation. The dots’ angular position on the circle were pseudorandomly chosen from a uniform distribution between 0 and 360 degrees under the constraint that any two dots could not overlap (minimum distance of 1.4° of visual angle). Colours of the three dots were taken from a continuous colour wheel with 100% saturation and 100% brightness (HSV) and a colour hue value randomly drawn from a uniform angular space (0 to 360 degrees), under the constraint of a minimum hue angular separation of 5° between the colours of the three dots, to avoid colours being indistinguishable.

### Procedure

The only difference between the first and second experiments was that the three coloured dots were presented simultaneously in the first experiment for a period of two seconds. In the second experiment, participants were presented with each dot for a period of 500ms followed by an inter-object blank of 500ms. In both experiments, the memory array was followed by a blank black screen of 4000ms (Fig. 1, **Presentation Phase**). After this delay, participants were probed in four different ways (Fig. 1, **Reproduction Phase**) - cued by colour, cued by location, or uncued, with either location first and then colour (L→C) or colour first and then location (C→L). In each case, the order of reporting objects was determined by the participant.

i) **Cued by colour**: Three squares (radius of 0.7° of visual angle) in the colours of the three original dots appeared centrally, each at a distance of 1.6 ° of visual angle from the screen centre (Fig. 1, **Reproduction Phase – Panel A**). These squares were equally spaced-out around the boundaries of this imaginary circle, starting from a random offset in a random order. Participants were asked to click on one of the squares, indicating which of the coloured objects they wanted to report. After they clicked on the chosen square, all squares disappeared, and a central white pentagon appeared at the screen centre. Throughout the whole experiment, this central pentagon indicated that participants could choose a location. A mouse cursor appeared at the screen centre. As soon as participants started moving the mouse, the pentagon disappeared and a dot in the colour that was selected appeared on the original imaginary circle at the angle the mouse cursor pointed towards, no matter whether the cursor was within or outside of the imaginary circle’s boundaries. The dot moved along the imaginary circle according to the mouse cursor’s angle from the screen centre, until a mouse button was clicked to confirm the location choice. After they clicked, participants went back to the screen on which they selected the first square. Now, however, the previously selected square was not shown, and participants selected from the two remaining squares. This process was repeated until all three locations were reported.
ii) **Cued by location**: Three dots were presented on the original imaginary circle in the original target locations, however, coloured in light grey (RGB [211 211 211], Fig. 1, **Reproduction Phase – Panel B**). The mouse cursor appeared at the screen centre. As soon as participants started moving the mouse, a cross appeared on the boundary of the circle at the angle the mouse cursor pointed towards, measured from the screen centre. Once the cross touched one of the three dots, participants could click a mouse button to confirm their location selection. After clicking, a colour wheel of 8.6° of visual angle appeared in the screen centre that was a visual representation of the colour space the stimuli colours were drawn from. The colour wheel was randomly rotated in each trial. As soon as participants started moving the cursor, the selected dot started changing its colour according to the angle from the screen centre in relation to the colour wheel. By clicking a mouse button, participants confirmed their colour choice. As in i), this procedure was repeated until all three colours were reported.
iii) **Uncued**,** location then colour**: Participants were presented with the pentagon that indicated they could choose a location by moving the mouse (Fig. 1, **Reproduction Phase – Panel C**) like in i). After they confirmed the location by clicking a mouse button, they were presented with the colour wheel and chose a colour for the chosen location, like in **ii)**. This was repeated two more times, so that subject produces both location and colour of all three objects.
iv) **Uncued**,** colour then location**: Participants were first presented with the colour wheel at the screen centre (see **ii** and **iii**) and a light-grey dot (RGB [211 211 211]) in its centre (Fig. 1, **Reproduction Phase – Panel D**). Once participants started moving the mouse, the central dot’s colour changed like in ii), and participants confirmed their colour selection by clicking the mouse button. Once they confirmed this selection, the pentagon appeared at fixation to indicate that they could choose its location. As in i), when the mouse moved, a dot of the chosen colour appeared on the original imaginary circle that moved according to the cursor’s position. This procedure was repeated until three dots were reproduced from memory.

### Analysis

All data and analysis scripts are available on OSF (https://osf.io/w39bh/?view_only=e52d4931fa3e4625a7fb87df92e9a9b4; *NOTE THAT THIS IS A VIEW-ONLY LINK FOR PEER-REVIEW WHICH WILL BE REPLACED BY THE STANDARD OSF LINK ON PUBLICATION*). The absolute error of recall was defined as the unsigned difference between target and response angles which is plotted in Supplementary Fig. 1.

### Probabilistic multifeature whole-report model

A novel Bayesian hierarchical model, based on the mixture model approach, was introduced to account for the sources of error in memory. The model estimates a categorical distribution across the possible orders in which participants report the three objects, and accounts for each response as a mixture of veridical responses, misbinding with one-feature reported from another object with a reciprocal swap, or an asymmetric misattribution. In these cases, the response is drawn from a von-Mises distribution around the fitting feature with precision representing the reciprocal of its variance. Complete guesses arise where a feature is *completely* forgotten and not reported for any of the objects. In this case, responses were drawn from a uniform distribution around the entire feature space. The model also accounts for three-way (cyclic) swaps, and the possibility that guesses for the two features within an object are correlated.

Unlike previous models, it also accounts for the joint distribution *across objects*, such that there is also a dependence of the colour response for object 1 upon the colour response for object 2, etc. Ultimately the model generates a 6-dimensional joint response distribution.

**Fig. 2:**
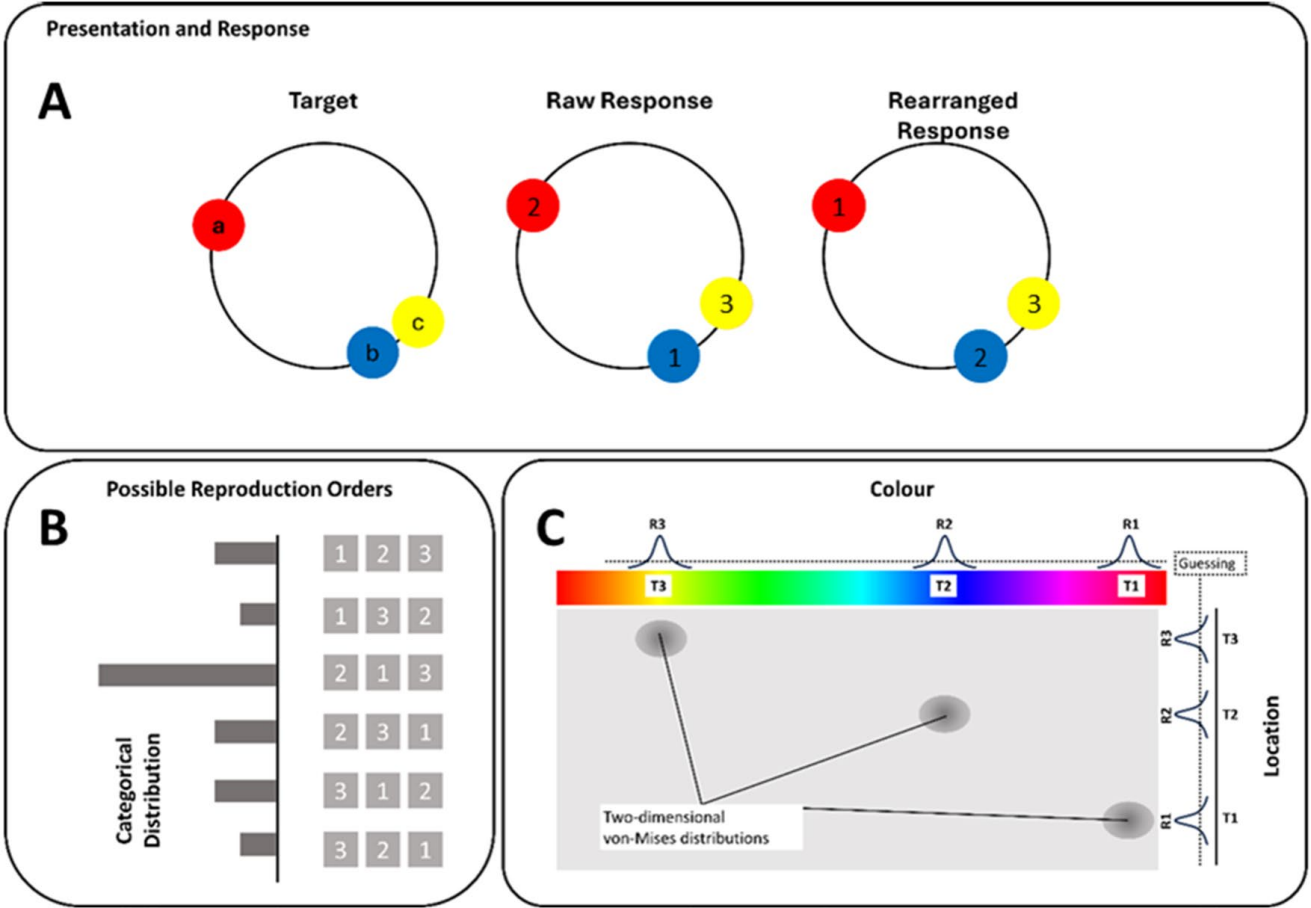
Schematic of the different steps run by the model. The original response stimuli (**A**) are rearranged according to a distribution drawn from a categorical distribution (**B**). Based on response (R) and target (T) pairs, responses are drawn from von-Mises distributions around their respective target colours and locations (**C**). If a response is counted as a guess, either of one or both features, the response is drawn from a uniform distribution around the whole response space.

Here we describe the generative model of participants’ responses, fitted by Gibbs sampling. In the multifeature whole-report condition, where participants reproduced stimuli in a self-selected order, the initial modeling step involved determining the response mapping. Since participants were free to choose their report sequence, the model considered all six possible permutations of the three objects (e.g., abc, acb, bac; see Fig. 2A). This selection process was modeled using a categorical distribution (the ‘rearranged response’ step; Fig. 2B), which identified the most likely latent identity for each response before analyzing the errors in feature space (Fig. 2C).

This step was essential to isolate genuine memory limitations from reporting strategies. Without the categorical mapping in Fig. 2B, the model would be forced to assume a fixed 1:1 correspondence (e.g., the first response always mapping to the first encoded object). Consequently, if a participant chose to report the third object first, the resulting large angular deviation would be incorrectly attributed to a memory failure –appearing as a guess or a misbinding (Supplementary Fig. 2) – rather than a simple shift in report order. By explicitly modeling this mapping, one can marginalize over the specific report order to determine the most likely origin of each feature. This ensures that the precision and guessing parameters in Fig. 2C accurately characterize the quality of visual working memory representations without being confounded by the arbitrary sequence of output.

Next, the model drew from a weighted categorical distribution whether participants made a swap error (P_swap_), a cyclic swap (P_cyclic_swap_) or neither (1 - P_swap_ - P_cyclic_swap_), introducing 2 free parameters. In symmetric swaps, participants exchange two features on the same dimension of two different objects for one another. Without a swap, location 1 (Fig. 1, **Error Types: L1**) was to be reported with colour 1 (Fig. 1, **Error Types: C1**), location 2 with colour 2 and location 3 with colour 3. If participants make a symmetric swap error, they report location 1 with colour 2 and, respectively, location 2 with colour 1 (Fig. 1, **Error Types: Symmetric Swap**).

Another way of forgetting is guessing, where a response is drawn from a uniform distribution. The model accounted for participants making guesses on either one (feature guess) or both features of an object (object guess) (2 free parameters). These were drawn from a Bernoulli distribution indicating whether the response was either drawn from a von-Mises or a uniform distribution (Fig. 2, **bottom right panel**).

In trials identified as involving swaps, we introduce an additional parameter to account for either an increased or decreased likelihood of guessing for one of the swapped objects. This means that, beyond the general guessing probability applied to all obects, we specifically model a different, potentially higher or lower, chance of guessing for the object involved in the swap. This added guessing term helps differentiate between general guessing errors and those specifically associated with objects that have been swapped, providing a more nuanced understanding of the errors occurring in these trials. Again, this could affect both features of the object, or just one of the two features (2 free parameters). Within the trials identified as swap trials, the model distinguishes whether the guessed feature/object is either part of the swap or is in the object otherwise unaffected by the swap (2 free parameters). I.e., referring to Fig. 1, the model assumes different guessing parameters for whether a feature or object guess occurred in L/C1 or L/C2 as opposed to L/C3 which was the unswapped object in the example. If the model samples a feature or object to be a guess, it draws the feature(s) from a uniform distribution (Fig. 2, **bottom right panel**). Otherwise, it draws them from a von-Mises distribution around the appropriate feature, with a given precision (2 free parameters, location and colour, Fig. 2, **bottom right panel**). The model assumes precision for colour and location to be identical for the three objects of each trial. Responses made later in a sequence have greater error due to ‘output interference’. This is true in both free recall^20–23^ and fixed recall orders^24,25^. However, here we are focused on which features were drawn from which objects, and we marginalised across output order.

This forward model generates some swaps without guesses, in which information about two objects is symmetrically exchanged, i.e. true, symmetric swapping (Fig. 1, **Error Types: Symmetric Misbinding**). However, in samples where swaps are accompanied by guesses within the swap, information is only exchanged on one side of the swap, and on the other side, there is a feature or a whole object guess, thus generating an asymmetric misattribution rather than a symmetric misbinding (Fig. 1, **Error Types: Asymmetric Misattribution**).

Note that the model presented here will identify a feature as a feature guess if a *correctly* reported feature was reported twice. In other words, should the participant be presented with the following set [C1:red, C2:blue, C3:yellow] and report [C1:red, C2:red, C3:yellow], the colour C2 would be counted as a feature guess if L2 was reported correctly. Hence, the model does not account for the possibility that participants may accurately recall a feature of an object even if they misreport it elsewhere, as would be expected in cases of retrieval errors. The model, thus, consisted of the following parameters:

1. *κ*_*color*_

2. *κ*_*loc​ation*_

3. *P*_*swap*_​

4. *P*_*cyclicswap*_​

5. *G*_*feature*_​

6. *G*_*object*_​

7. *G*_*feat_swap_non*_​

8. *G*_*feat_swap_swp*_​

9. *G*_*obj_swap_non*_​

10. *G*_*obj_swap_swp​*_

11. *G*_*feat_cyclic*_​

12. *G*_*obj_cyclic*_​

13. *Reproduction Order*

These 13 free parameters were estimated per subject, hierarchically constrained by a population mean and variance, using maximum likelihood. Each parameter was allowed to vary between cued trials and the *C→L* and *L→C* uncued conditions (See Model Parameters in Supplementary Material for details,** see** Table 1**for a summary of error types**). Sampling used 5000 adaptation samples, with 10,000 iterations, in 4 chains, resulting in a total of 40,000 samples. Means for each participant were computed over these 40,000 samples. It took about 26 h of compute time on an Intel i7920X CPU @2.90 GHz with 32 GB RAM to fit the full model to data from one experiment.

Model comparison was conducted using the Watanabe-Akaike Information Criterion (WAIC;^26^. WAIC is a fully Bayesian approach for estimating the out-of-sample predictive accuracy of a model. Unlike the Deviance Information Criterion (DIC) or the Akaike Information Criterion (AIC), WAIC utilizes the entire posterior distribution rather than a single point estimate, making it more robust for hierarchical models with complex parameter spaces^27^. To facilitate comparison, we also reported Δ WAIC values, defined as the difference between a given model’s WAIC and the WAIC of the best-fitting model (the model with the lowest absolute WAIC). A ΔWAIC of zero identifies the most fitting model; higher values indicate decreasing evidence for a model relative to the best-fitting candidate.

**Table 1:**
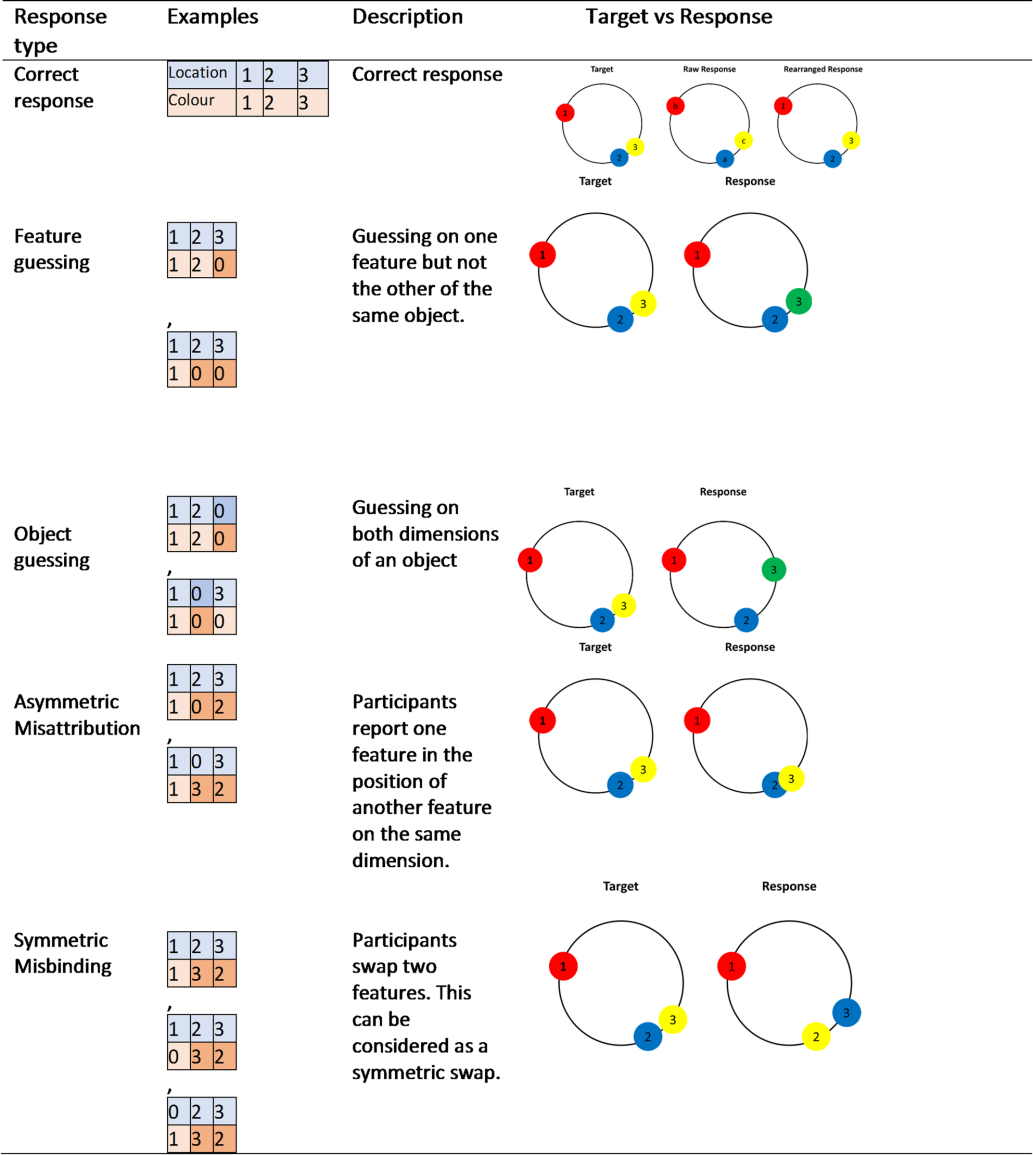
Examples for the different response types.

Note: While numbers 1 to 3 indicate the appropriate object the von-Mises distribution is drawn from, 0 indicates that the response is drawn from a uniform distribution around the response space (**see** Fig. 2).

To quantify the strength of evidence for the comparisons conducted, Bayes Factors (BF_10_) were calculated using a default Cauchy prior of 0.707. The BF_10_ represents the ratio of the likelihood of the data under the alternative hypothesis H_1_ relative to the null hypothesis H_0_. Resulting values were interpreted according to the following, commonly applied classification: values of BF_10_ > 100 were considered extreme evidence for H_1_; 30–100 as very strong; 10–30 as strong; and 3–10 as moderate. Values between 1 and 3 were treated as anecdotal or inconclusive, indicating that the data did not strongly support one hypothesis over the other. Conversely, BF_10_ values below 1/3 were interpreted as evidence in favour of the null hypothesis H_0_, with BF_10_ < 0.01 representing extreme evidence for H_0_.

## Results

### EXPERIMENT 1: free multifeature whole-report of simultaneously presented stimuli

#### Mixture modelling confirms co-existence of multiple error types beyond symmetric misbinding

The full model had a better fit (WAIC = 18183.57, ΔWAIC = 73.15) than the null model without guessing or swaps (WAIC = 29080.26, ΔWAIC = 18110.42). The full model also showed a better fit than a model that does not allow for guesses given a swap and therefore does not allow for asymmetric misattribution (WAIC = 33561.61, ΔWAIC = 15,451.19). Further, calculating WAICs for a model not accounting for a difference between free and cued recall (WAIC = 18268.01, ΔWAIC = 157.59) and a model not accounting for whether colour or location was first at probe (WAIC = 18110.42, ΔWAIC = 0) showed that the latter was the best fitting model.

**Fig. 3:**
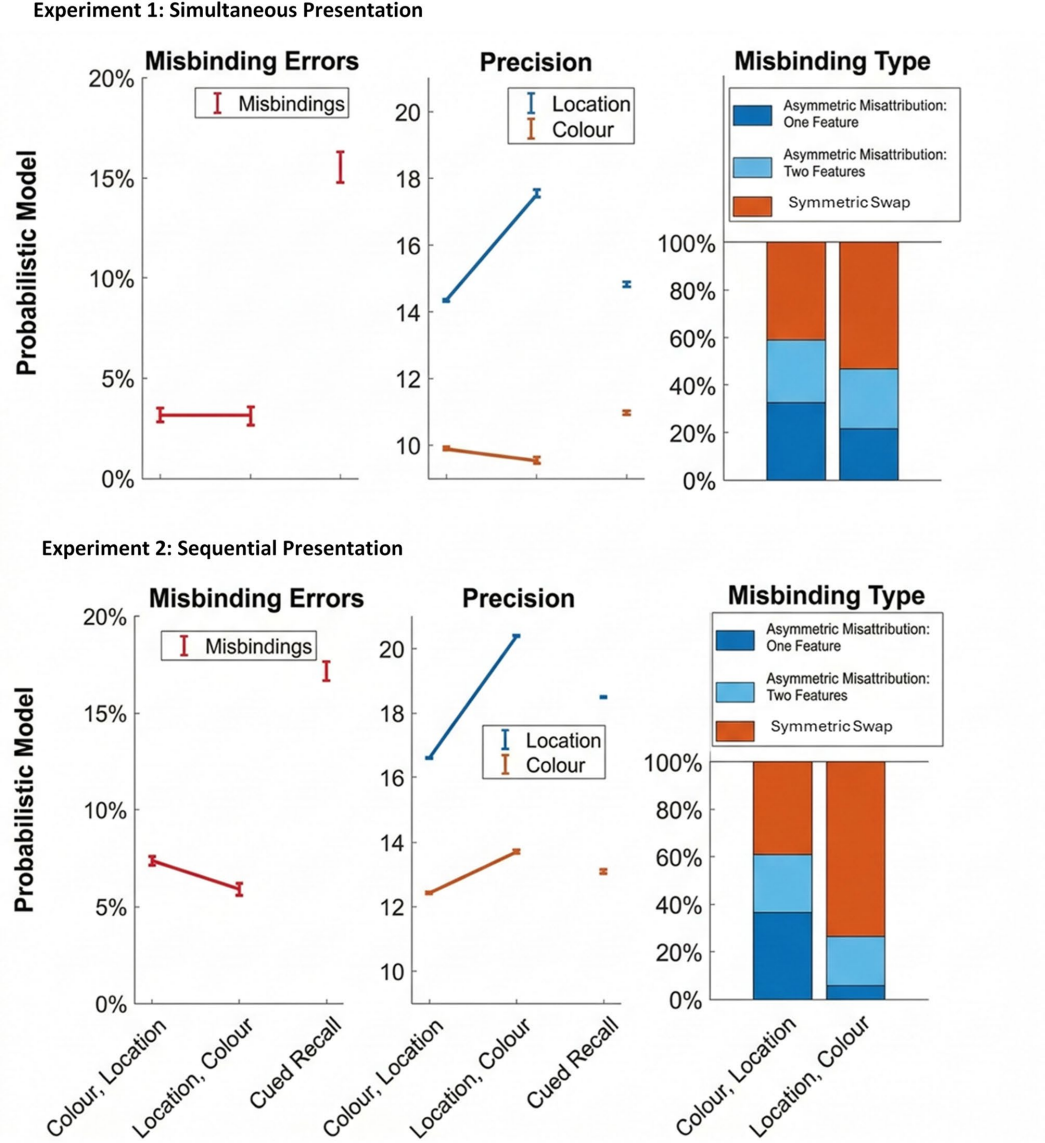
Hierarchical Model results for Experiments 1 (top) and 2 (bottom). Errors identified as swap errors (left), precision (middle) and actual types of swap errors (right). Swapping was increased in cued conditions. The model further allowed us to distinguish between symmetric misbinding and asymmetric misattribution. There was strong evidence for the co-existence of symmetric misbinding and asymmetric misattribution. In the *Colour First* condition, participants were significantly more precise for colour, yet they produced more swap errors than in the *Location First* condition. Asymmetric misattribution occurred more often in the *Colour First* condition. Error bars represent within-subject standard errors of the mean (SEM) calculated using the normalization procedure described by Loftus and Masson (1994). To isolate within-subject variance, each participant’s mean performance across all conditions was subtracted from their individual per-condition values prior to calculating the error estimate. (Loftus & Masson, 1994).

We found strong evidence for both symmetric misbinding and asymmetric misattribution errors (Fig. 3, **Probabilistic Model**). Across the cued conditions, symmetric misbding errors had an estimated probability of 15.4% (SD = 5.81%). In the cued condition, we know with confidence which object the cued feature belongs to. This means that symmetric misbinding errors are naturally estimated as higher and are not directly comparable with the uncued conditions. This is because if there is guessing on the cued dimension, then a situation where one dimension is forgotten and the other is remembered correctly (i.e. a *feature* guess) is incorrectly labelled as a symmetric misbinding error. Thus, the common way of calculating symmetric misbinding errors (from report of a single feature) might lead to inflated values in comparison to swaps measured in a full report of all features presented.

In the uncued conditions where participants were either to report location and then colour (L→C) or vice versa (C→L), *swaps* accounted for 2.99% (SD = 1.31%, C→L) and 2.91% (SD = 1.49%, L→C), with moderate evidence for the absence of a difference (BF_10_ = 0.26). Within these, 42.8% (SD = 5.95%; C→L) and 62.9% (SD = 5.54%; L→C) of *swaps* were symmetric misbinding. Reproducing the location first increased symmetric misbinding with extreme evidence in the paired-sample Bayesian t-test (BF_10_ = 7.06e + 25), suggesting a directionality in binding between locations and colours.

*Swaps* could be identified as asymmetric misattributions either due to forgetting of just one feature (feature guess) or due to forgetting of two features (object guess) and thus the whole object. Of all trials initially identified as swaps, 29.8% (SD = 6.16%; C→L) and 13.4% (SD = 2.95%; L→C) were asymmetric misattributions due to feature guessing and 27.4% (SD = 1.46%; C→L) and 23.7% (SD = 2.61%; L→C) were asymmetric misattributions due to forgetting of one whole object. Here, paired-sample t-tests showed extreme evidence that asymmetric misattributions due to feature or object forgetting were both *less* frequent when location had to be reported first (BF_10_ = 1.63e + 12 asymmetric misattribution due to feature guessing, and BF_10_ = 923.00 asymmetric misattribution due to object guessing).

Location showed highest precision when reported first, thus, precision for location was highest in L→C (17.6, SD = 5.34) followed by the cued condition (14.8, SD = 4.94) and C→L (14.3, SD = 5.03). Bayes factors suggest extreme evidence for differences between all three conditions (L→C vs. cued: BF_10_ = 7.06e + 9; C→L vs. cued: BF_10_ = 1.17e + 7; L→C vs. C→L: BF_10_ = 3.06e + 12). On the other hand, precision for colour followed the opposite pattern. i.e., it was highest in the cued condition (10.9, SD = 2.70), followed by C→L (9.96, SD = 2.69) and L→C (9.66, SD = 2.81). Bayes factors showed extreme evidence for the difference between the cued and whole-report conditions and almost moderate evidence for the difference between the whole-report conditions (cued vs. C→L: BF_10_ = 2.47*10^16^, cued vs. L→C: BF_10_ = 1.28*10^6^; C→L vs. L→C: BF_10_ = 2.97).

An overview of these findings is available in Table 2, prior-sensitivity analysis is available in Supplementary Fig. 3. In summary, there was not only evidence for the co-existence of symmetric misbinding and asymmetric misattribution errors but their contribution to the overall number of trials initially identified as swaps is impacted on by the order in which participants are to reproduce the features of an object. However, the WAIC generally prefers a model that does not make a difference between the two uncued conditions. Cyclic swaps only accounted for a small proportion of errors.

In the conditions without a cue, there was a reporting bias towards reporting the topmost object first in 59.81% of cases across participants and conditions and also to report the object that was presented furthest to the left, suggesting that in the absence of external guidance, participants defaulted in their reporting to a strategy that might be shaped by reading habits (top-to-bottom and left-to-right).

#### Cued recall yields higher estimates of swap errors

Finally, we examined the effect of showing participants the possible values of one feature as a cue (instead of requiring recall), comparing the overall absolute error (Supplementary Fig. 1). We expected cues to improve recall, but in fact they worsened recall. Histograms of raw reporting error are available in Supplementary Fig. 4.

**Table 2:** Comparison of errors between the colour first and location first conditions in experiment 1.

Parameter	C→L (Mean ± SD)	L→C (Mean ± SD)	Evidence (BF_10_​)	Interpretation
Overall probability of swaps	2.99% ± 1.31%	2.91% ± 1.49%	0.26	Moderate evidence for H0​
Symmetric misbinding	42.8% ± 5.95%	62.9% ± 5.54%	7.06 × 10^25^	Extreme evidence for H1​ (L→C > C→L)
Asymmetric: feature guessing	29.8% ± 6.16%	13.4% ± 2.95%	1.63 × 10^12^	Extreme evidence for H1​ (C→L > L→C)
Asymmetric: object guessing	27.4% ± 1.46%	23.7% ± 2.61%	923	Extreme evidence for H1​ (C→L > L→C)
Precision for location	14.3 ± 5.03	17.6 ± 5.34	3.06 × 10^12^	Extreme evidence for H1​ (L→C > C→L)
Precision for colour	9.96 ± 2.69	9.66 ± 2.81	2.97	Anecdotal evidence for H1​

### EXPERIMENT 2: sequentially presented stimuli

#### Evidence for asymmetric misattribution in the sequential experiment

We fitted the Mixture Model to responses. As for Experiment 1, the full model had a better fit (WAIC = 7833.73, ΔWAIC = 39.18) than the null model without guessing or swaps (WAIC = 17479.31, ΔWAIC = 9684.76). The full model also showed a better fit than a model that does not allow for guesses given a swap and therefore does not allow for asymmetric misattributions (WAIC = 22130.43, ΔWAIC = 14335.88). Further, a model not accounting for a difference between free and cued recall (WAIC = 7839.82, ΔWAIC = 45.27) and a model not accounting for whether colour or location was first at probe (WAIC = 7794.55, ΔWAIC = 0) showed that the latter was the overall best fitting model.

Reproduction order output is available in the supplementary materials.

Confirming results of the first experiment, there was extreme evidence that precision for location was higher in the L→C compared to the C→L condition (20.36 (SD = 4.34) vs. 16.65 (SD = 4.25); BF_10_ = 8.17e + 20) and Cued condition (18.45 (SD = 4.22); BF_10_ = 9.60e + 14). There was also extreme evidence that it was higher in the C→L compared to the Cued condition (BF_10_ = 1.72e + 23).

In this experiment, precision for colour follows the same pattern as precision for location, that is, there was extreme evidence that it was higher in the L→C compared to the C→L condition (13.75 (SD = 3.36) vs. 12.49 (SD = 3.29); BF_10_ = 3.83e + 13) and Cued condition (13.06 (SD = 3.24); BF_10_ = 10734.28). It was also higher in the Cued compared to the C→L condition, supported by extreme evidence (BF_10_ = 58008.73).

Swap errors occurred at a frequency of 17.11% (SD = 7.04%) in the cued condition. Again, extreme evidence suggested that participants made fewer swap errors in the L→C condition compared to the C→L condition (5.87% (SD = 4.02%) vs. 7.35% (SD = 4.63); BF_10_ = 16299.68).

#### More symmetric misbinding when reporting location first

There was extreme evidence in support of more symmetric misbindings in L→C (68%, SD = 6.88%) than in C→L (37.6%, SD = 11.9%; BF_10_ = 2.11e + 12) but fewer asymmetric misattributions with guessing on one feature (L→C: 9.54%, SD = 2.29; C→L: 39.2%, SD = 9.07%; BF_10_ = 2.57e + 10). Bayesian analysis provided anecdotal evidence for the absence of a difference between the two uncued conditions in terms of asymmetric misattribution based on guessing of the whole object (C→L: 23.2%, SD = 2.84% vs. L→C: 22.5%, SD = 4.62%; BF_10_ = 0.76).

In the sequential experiment (Fig. 3, bottom), reporting colour first produces more trials identified as asymmetric misattribution with feature guessing with lower precision, in comparison to reporting *Location First* (Table 3, prior-sensitivity analysis is available in Supplementary Fig. 5), indicating that location might be asymmetrically bound to other features and thus confirming earlier findings from Heuer et al.^10^ and Schneegans et al.^11^. Again, cyclic swaps only accounted for a small proportion of errors.

Absolute error for this experiment is available in Supplementary Fig. 3, histograms of raw response errors are available in Supplementary Fig. 6. Further analysis on the preferred reproduction order is available in the Supplementary Materials.

**Table 3:** Comparison of errors between the colour first and location first (uncued) conditions in experiment 2.

Parameter	C→L (Mean ± SD)	L→C (Mean ± SD)	Evidence (BF10​)	Interpretation
Overall probability of swaps	7.35% ± 4.63%	5.87% ± 4.02%	16299.68	Extreme evidence for H1​ (C→L > L→C)
Symmetric misbinding	37.6% ± 11.9%	68% ± 6.88%	2.11 × 10^12^	Extreme evidence for H1​ (L→C > C→L)
Asymmetric: feature guessing	39.2% ± 9.07%	9.54% ± 2.29%	2.57 × 10^10^	Extreme evidence for H1​ (C→L > L→C)
Asymmetric: object guessing	23.2% ± 2.84%	22.5% ± 4.62%	0.76	Anecdotal evidence for H0​
Precision for location	16.65 ± 4.25	20.36 ± 4.34	8.17 × 10^20^	Extreme evidence for H1​ (L→C > C→L)
Precision for colour	12.49 ± 3.29	13.75 ± 3.36	3.83 × 10^13^	Extreme evidence for H1​ (L→C > C→L)

#### Simultaneous encoding promotes asymmetric misattribution in the absence of cues, while sequential encoding improves precision

For systematic comparison of the two experiments, we collapsed over conditions C→L and L→C with unpaired t-tests (Table 4, prior-sensitivity analysis in Supplementary Fig. 8). There was anecdotal evidence for a difference in trials identified as including a swap error (BF_10_ = 0.41) in the cued conditions and only anecdotal evidence for a difference between the experiments in the uncued trials (BF_10_ = 2.85). Within these trials, the proportion of symmetric misbinding and asymmetric misattribution due to forgetting of one feature showed to be inconclusive between experiments with anecdotal evidence for the absence of a difference (BF_10_ = 0.93 and, respectively, BF_10_ = 0.40). However, simultaneous presentation of objects increased levels of asymmetric misattribution due to object guessing, supported by moderate evidence (BF_10_ = 7.35). On the other hand, there was extreme evidence that simultaneous presentation increased the general, baseline rate of object guessing (BF_10_ = 82830.90).

There was moderate evidence that precision for location was slightly higher with sequential encoding for the cued condition (BF_10_ = 3.09), with anecdotal evidence that there was no evidence for the uncued conditions (BF_10_ = 0.97). Precision for colour was overall higher with sequential encoding, supported by anecdotal and strong evidence (Cued: BF_10_ = 2.07; Uncued: BF_10_ = 24.18).

Further, when data was collapsed over both experiments, across individuals, there was extreme evidence that the swap rate was positively correlated with the general, baseline object guessing level (*r* = 0.62, BF_10_ = 1089.73), suggesting that participants who make more errors of symmetric swapping and asymmetric misattribution also show increased levels of guessing for the whole object. We did not observe such correlations between the swap rate and any of the other guessing rates (Supplementary Table 1), nor with participants’ precision, or with the composition of the errors.

**Table 4:** Comparison of errors between the colour first and location first (uncued) conditions in experiment 2.

Parameter	Simultaneous	Sequential	Evidence (BF10​)	Interpretation
Swaps: cued	15.4% ± 5.81%	17.11% ± 7.04%	0.41	Anecdotal evidence for H0​
Swaps:	3.89% ± 1.90%	6.40% ± 4.23%	2.85	Anecdotal evidence for H1 (Seq. > Sim.)​
Symmetric	52.13% ± 5.14%	55.95% ± 8.74%	0.93	Anecdotal evidence for H0​
Asymmetric due to feature guess	22.27% ± 4.45%	21.10% ± 4.93%	0.4	Anecdotal evidence for H0​
Asymmetric due to object guess	25.60% ± 1.15%	22.96% ± 3.88%	7.35	Moderate evidence for H1​ (Sim. > Seq.)
Colour precision				
Cued	10.90 ± 2.70	13.06 ± 3.24	2.07	Anecdotal evidence for H1​
Uncued	9.75 ± 2.73	13.10 ± 3.29	24.18	Strong evidence for H1​ (Seq. > Sim.)
Location precision				
Cued	14.80 ± 4.94	18.45 ± 4.22	3.09	Moderate evidence for H1​ (Seq. > Sim.)
Uncued	15.91 ± 5.18	18.51 ± 4.28	0.97	Anecdotal evidence for H0​ (Inconclusive)

## Discussion

Short term memory recall errors often occur due to “misbinding”, where features are reported from the wrong object. Here we asked whether these errors are true swaps, where two features of two objects are symmetrically exchanged, or whether they are in fact asymmetric misattributions, with only one feature transposed to the wrong object. Participants had to reproduce colours and locations of three dots with or without a cue. Without a cue, participants were required to reproduce all features on all dimensions (whole report) of all objects in the memory array (whole report). This enabled us to examine the fate of every single remembered feature. In order to allow to investigate effects of feature binding across a whole array, we introduce a new Bayesian hierarchical mixture model for free multifeature whole-report errors. The model showed that what is commonly labeled misbinding includes both symmetric swap errors and asymmetric misattributions. Asymmetric misattributions can be modelled as a “swap” with forgetting of one feature or both features of one object of the pair. Within our dataset, asymmetric misattribution account for almost 50% of errors composing of misbinding and misattribution. Yet, these error types are generally rare – around 5% of trials -- when all the features must be reported.

Simultaneous presentation of objects increased the tendency to asymmetric misattribution. For example, when the colour of one object was reported at the wrong position, the correct position was often reported with a guessed colour. This effect of simultaneity suggests that swapping one feature while forgetting the other feature of an object is dependent on encoding, and thus might be coupled to perception.

Performance was worse when participants had to reproduce the colour first. This could support the privileged binding of objects to locations, in line with some previous work^9–11^. The direct comparison between the first and second experiment further suggests simultaneous encoding promotes asymmetric misattribution. When all objects are presented simultaneously, these asymmetric misattributions, in which information is only exchanged from one objects to the other but not reciprocally, occur slightly more often. On the other hand, the precision for colour was increased by sequential encoding. A possible explanation for this finding might be an increased encoding time per object. The fact that some model parameters improve, and some worsen in sequential over simultaneous experiments and vice versa sheds new light on the ambiguous literature on difference between sequential and simultaneous presentation^28,29,30^.

We included a condition in which one dimension was already given. This enabled us to compare performance with standard cued recall. Our data demonstrate that simply knowing the possible values of the cue dimension can affect recall^33^. However, the parameters from our cued and uncued trials cannot be directly compared. For example, misbinding appears artificially higher with a cue, since one dimension of each object is faithfully provided. The precision results, however, were qualitatively the same whether or not cued trials were included in the analysis.

The distinction between swap errors as genuine perceptual errors and as potential guessing strategies is crucial but not fully resolved in our study. While swap errors involve misreporting features between objects, these could occur at the time of encoding or could reflect strategic guessing when memory is uncertain. While our model differentiates these errors, further studies are needed to clarify the underlying cognitive processes.

Future studies should incorporate confidence ratings to distinguish high-confidence perceptual errors from low-confidence guessing. Additionally, manipulating the perceptual similarity of objects or the complexity of memory arrays could help determine whether swap errors are more perceptual or strategic in nature. These approaches will in the future improve theoretical models in visual short-term memory.

Incorporating confidence ratings into future analyses could provide valuable insights into the nature of swap errors. One would predict that high-confidence responses will likely indicate genuine perceptual errors, while low-confidence responses may correspond with higher instances of swap errors and feature guessing, suggesting strategic guessing when memory is uncertain. This differentiation can help clarify whether symmetric misbinding and asymmetric misattribution errors, and particularly the latter, are primarily due to perceptual misbinding or are influenced by uncertainty-driven guessing.

While the multifeature whole-report paradigm provides new insights into the binding structure of visual working memory, this approach involves methodological trade-offs. Most notably, requiring participants to sequentially report multiple features across all objects could inadvertently increase the potential for output interference compared to standard single-item cued recall. In the presented modeling approach, we explicitly marginalized across report order to isolate the structural sources of error in order to specifically distinguish symmetric swaps from asymmetric misattributions. Consequently, the absolute error rates observed here may be influenced by the cumulative cognitive load of sequential reporting and the additional time passing between presentation and reporting the last feature. I.e., the model cannot rule out that the correctly (but misattributed) color of one object is reported first and the other (forgotten) color would have been reported second or third, but is lost by that time and must therefore be guessed.

Finally, regarding model comparisons, results showed that while Bayes Factors provided strong evidence for localized parameter differences between uncued conditions (such as varying rates of asymmetric misattributions), the WAIC generally favored a simpler model that collapsed across these conditions. This could reflect the WAIC’s strict penalization of global model complexity when adding parameters to capture specific, localized effects, yet parameter mimicry cannot be fully ruled out.

Another intriguing question is whether misbinding in the experiment represents a primary failure of encoding or a secondary failure during retrieval: It is possible that some errors identified as asymmetric were originally stored as symmetric swaps, but the reciprocal part of the swap was lost due to output interference. That is, the participant might successfully report the first misattributed feature, but the cognitive load of this report causes the remaining features of the swapped pair to be forgotten, leading to a guess.

While the presented model marginalizes over report order, this ‘swap *then* forget’ account remains a viable alternative error type. If output interference were the primary driver, one might expect the proportion of asymmetric errors to increase significantly for objects reported later in the sequence. Future research could explicitly test this by manipulating the required report order.

The unique probing method introduced here, together with the generative model we describe, allows for the first time direct assessment of the full binding structure in short term memory. This measures feature binding over a whole memory array, making it possible to distinguish between new kinds of memory error. It opens up analysis of new aspects of visual short term memory to future empirical enquiry. Our model code is openly available with instructions (https://osf.io/w39bh/overview). Such a design could allow future assessment of how factors such as delay or set size impact feature binding, as well as disease processes that affect memory.

## Supplementary Information

Below is the link to the electronic supplementary material.

Supplementary Material 1

## Data Availability

All data and analysis scripts are available on OSF (https://osf.io/w39bh/overview).
