# Supplementary material for: Evidence from multifeature whole-report in visual short-term memory suggests that not all misbinding is swapping
Source: Sci Rep. 2026 May 23;16:16012. doi: 10.1038/s41598-026-52649-7 (PMC13198544; doi:10.1038/s41598-026-52649-7)
Supplement: Supplementary file 1 — Supplementary Material 1 [file 41598_2026_52649_MOESM1_ESM.docx]

**Supplementary materials**

Simplified Code for Bayesian model

# 1. LATENT REPORTING STRATEGY

# Determines which of the 6 possible object-to-response mappings (e.g., ABC, BAC)

# the participant used, to avoid confounding report order with memory error.

ReproductionOrder ~ dcat(c(1/6, 1/6, 1/6, 1/6, 1/6, 1/6))

# 2. ERROR TYPE SELECTION

# Categorizes the trial into one of three types:

# 1: No Swap, 2: Symmetric Swap (2-way), 3: Cyclic Swap (3-way)

swaptype ~ dcat( 1 - P_swap - P_cyclicswap, P_swap, P_cyclicswap )

# If a swap is selected, determine which specific objects are involved (e.g., objects 1 & 2)

Swap_position ~ dcat(c( 1/3, 1/3, 1/3 ))

Cycl_swap_position ~ dcat(c( 1/2, 1/2))

# 3. GUESSING MECHANISMS

# Determines if information for a specific feature or the entire object was lost.

Feature_Guess ~ dbern(G_feature_guess) # Probability of forgetting one feature

Object_Guess ~ dbern(G_object_guess) # Probability of forgetting the whole object

# Parameters specific to swap trials to detect Asymmetric Misattribution.

# If within a swap a feature or the whole object is 'guessed', it becomes asymmetric.

Feature_Guess_Swap ~ dbern(G_feature_guess_swap)

Object_Guess_Swap ~ dbern(G_object_guess_swap)

# 4. FINAL RESPONSE GENERATION

# If 'remembered' == 1: Response is centered on the target with learned precision.

# If 'remembered' == 0: Response is drawn from a Uniform distribution (0, 2π).

Response ~ dvonmises(Target, remembered * precision)

where ‘remembered’ is 1 or 0 defining whether the response is drawn from a von mises distribution around the target, or from a uniform distribution in circular space.


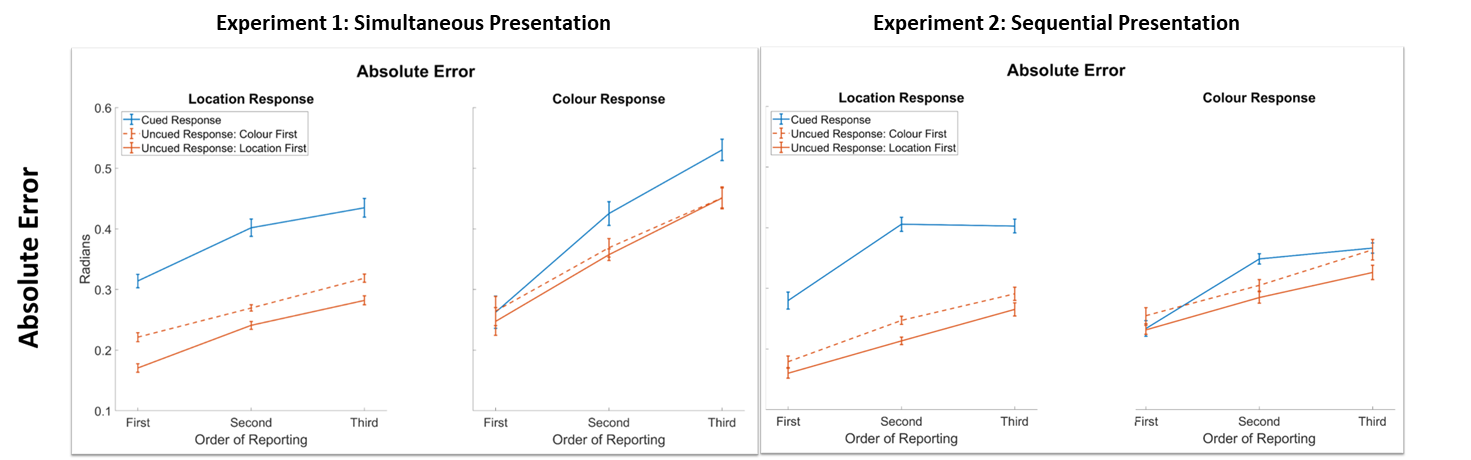


**Supplementary Figure 1 | Absolute error in radians between response and target for location (left panel) and colour recall (right panel) conditions in Experiment 1 (left half) and Experiment 2 (right half).** Participants performed better in uncued compared to cued conditions. This benefit was greater for location responses. There was also a benefit for when the location was reported first.

**Supplementary Figure 2 | Impact of Response Rearrangement on Model Results.** Targets (T1-T3) are presented on the left and raw responses on the right (R1-R3). In this simplified example, the participant correctly reports all objects, yet the order of responses is not identical to the order of targets. Panels illustrate how the "rearranged response" step, where the sequence is re-ordered based on the probabilistic model leads to a “correct” response (green), while the raw order would lead to misbinding.


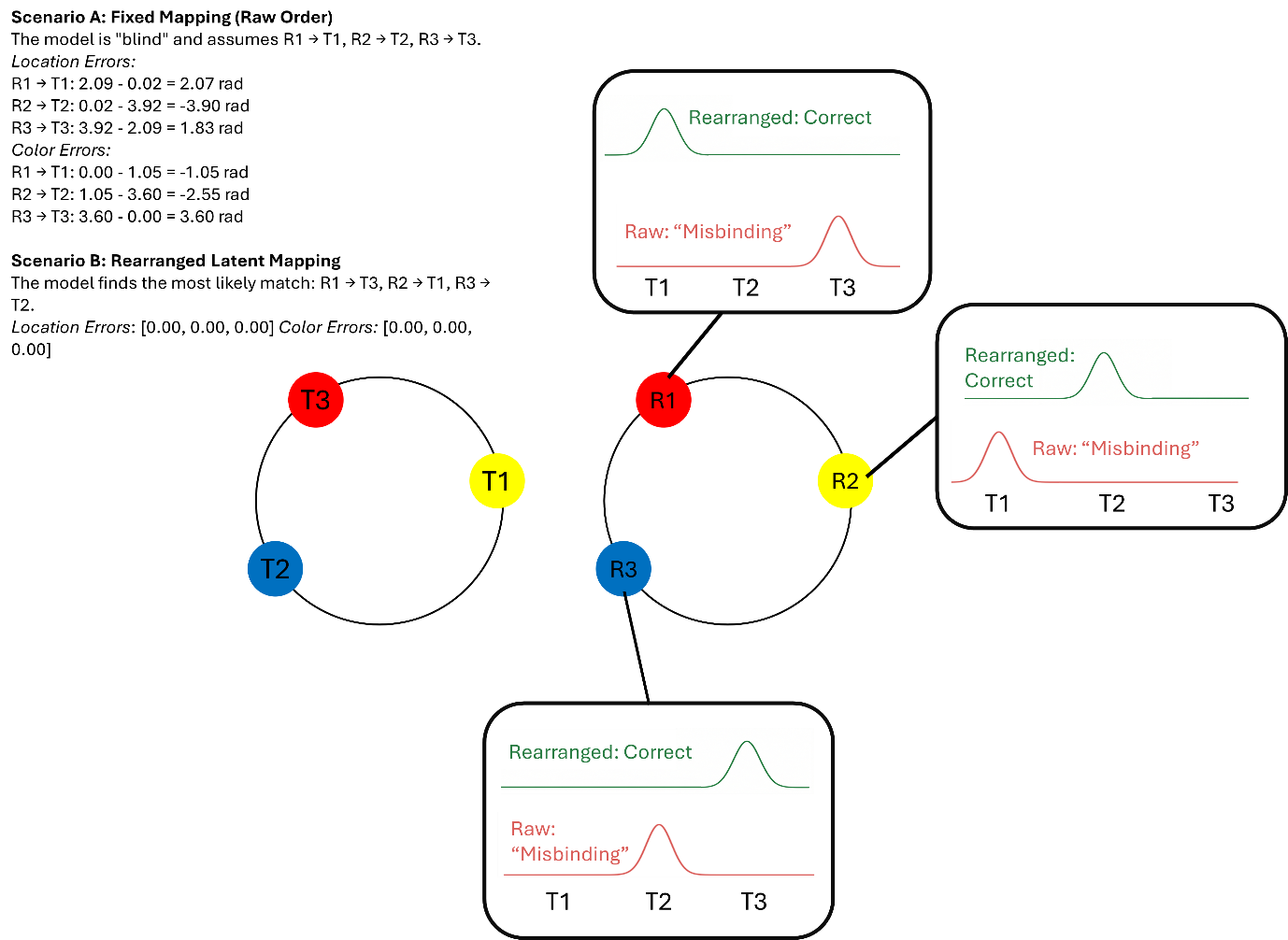


**Supplementary Figure 3 | Bayesian Prior Robustness Analysis for Experiment 1.** The plot illustrates the sensitivity of the Bayes factor BF_10_ to the choice of the prior distribution width for the effect size under the alternative hypothesis H1. The x-axis represents the scale parameter (width) of the Cauchy prior distribution, and the y-axis represents the resulting Bayes factor.


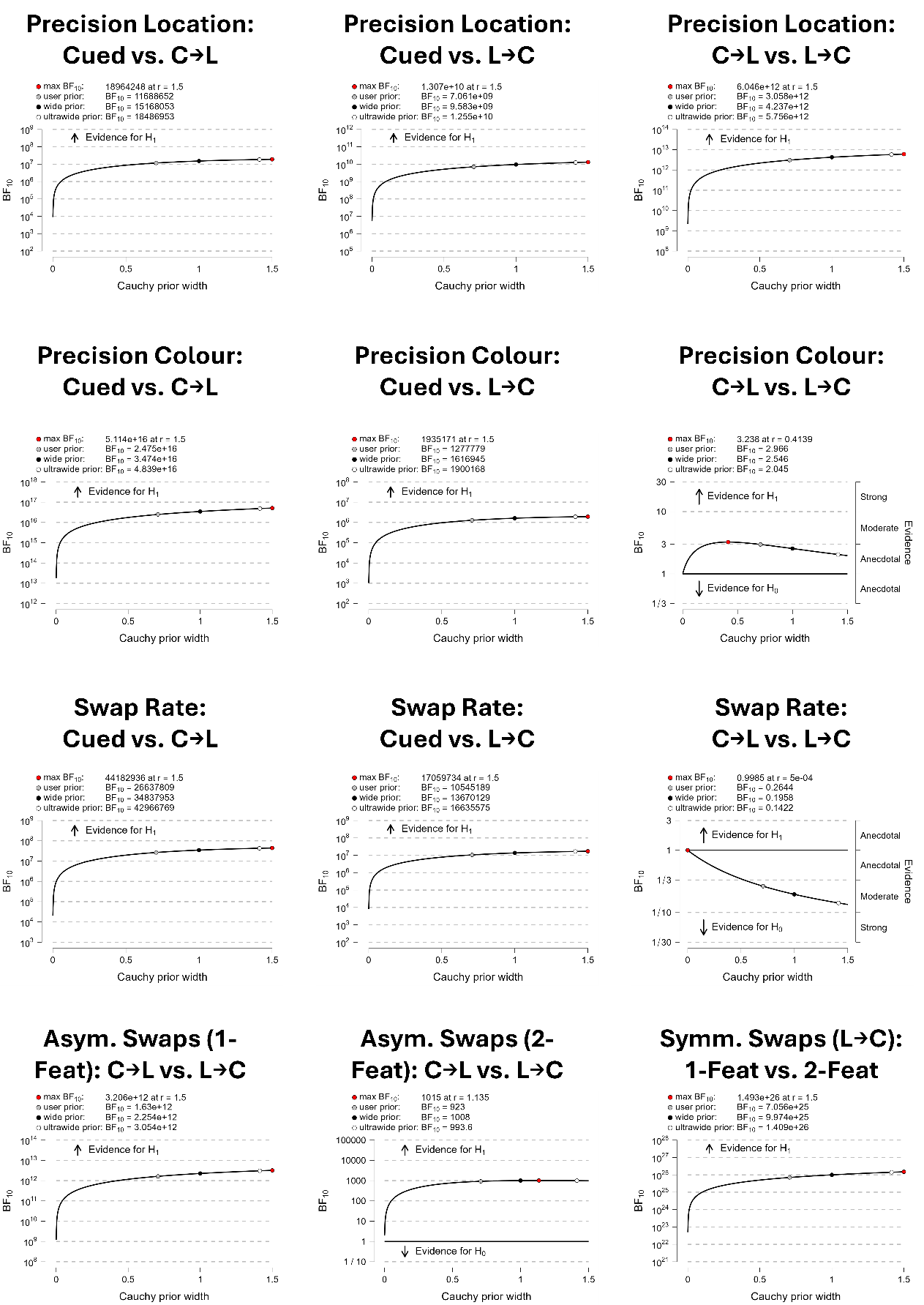


**Supplementary Figure 4 | Raw response error distributions for Experiment 1 across report order.** Histograms illustrate the frequency of response errors (calculated as the angular deviation between the target value and the reported value in radians) for Location and Colour. The data are collapsed across participants but separated by report order (1st, 2nd, and 3rd response) and by condition (L🡪C, C🡪L, cued recall).


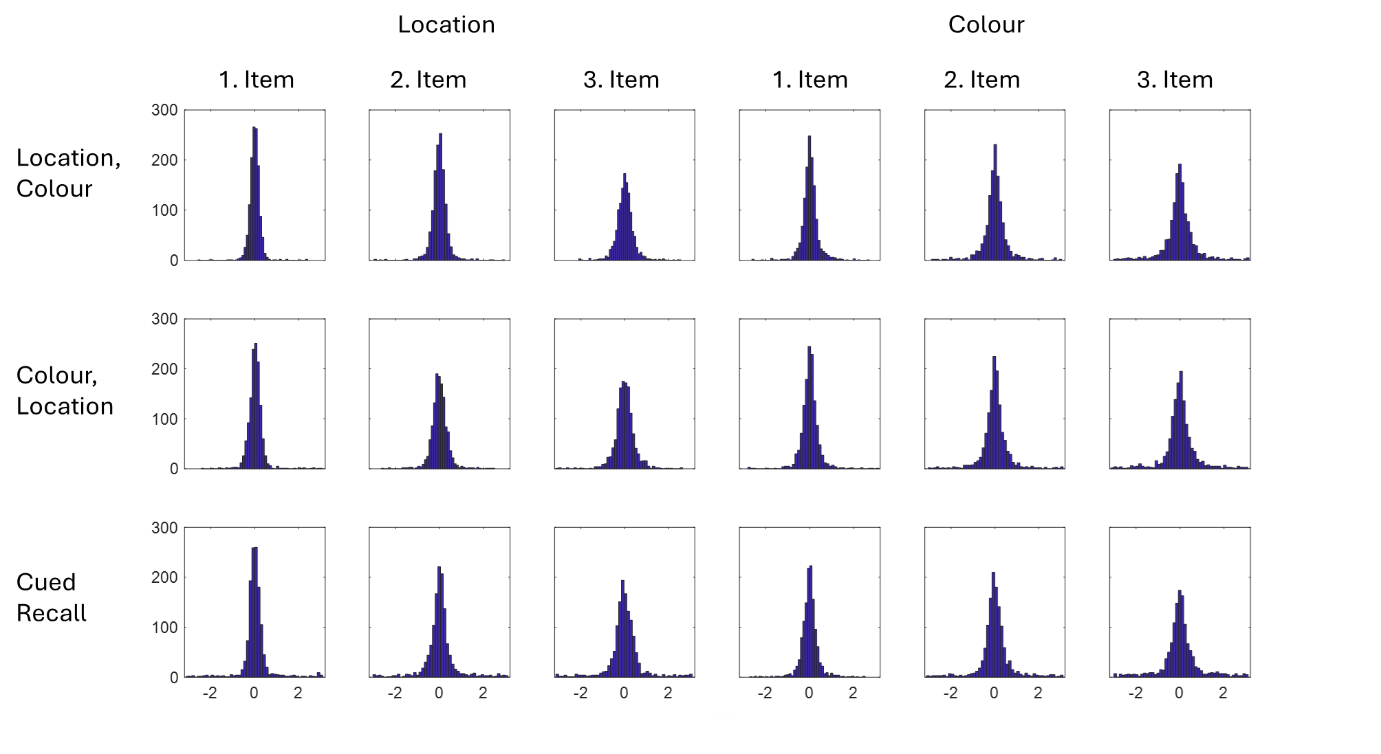


**Supplementary Figure 5 | Bayesian Prior Robustness Analysis for Experiment 2.** The plot illustrates the sensitivity of the Bayes factor BF_10_ to the choice of the prior distribution width for the effect size under the alternative hypothesis H1. The x-axis represents the scale parameter (width) of the Cauchy prior distribution, and the y-axis represents the resulting Bayes factor.


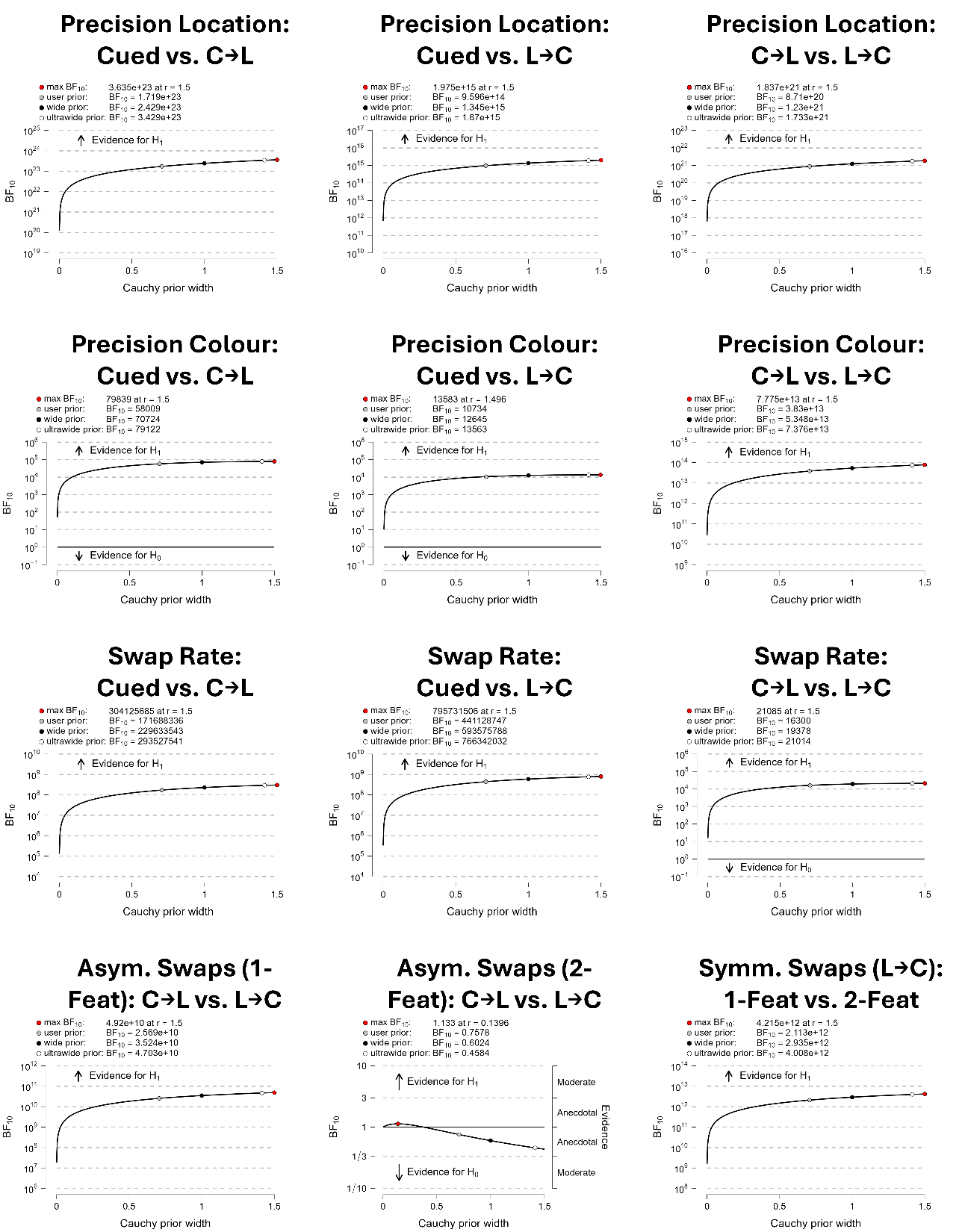


**Supplementary Figure 6 | Raw response error distributions for Experiment 2 across report order.** Histograms illustrate the frequency of response errors (calculated as the angular deviation between the target value and the reported value in radians) for Location and Colour. The data are collapsed across participants but separated by report order (1st, 2nd, and 3rd response) and by condition (L🡪C, C🡪L, cued recall).


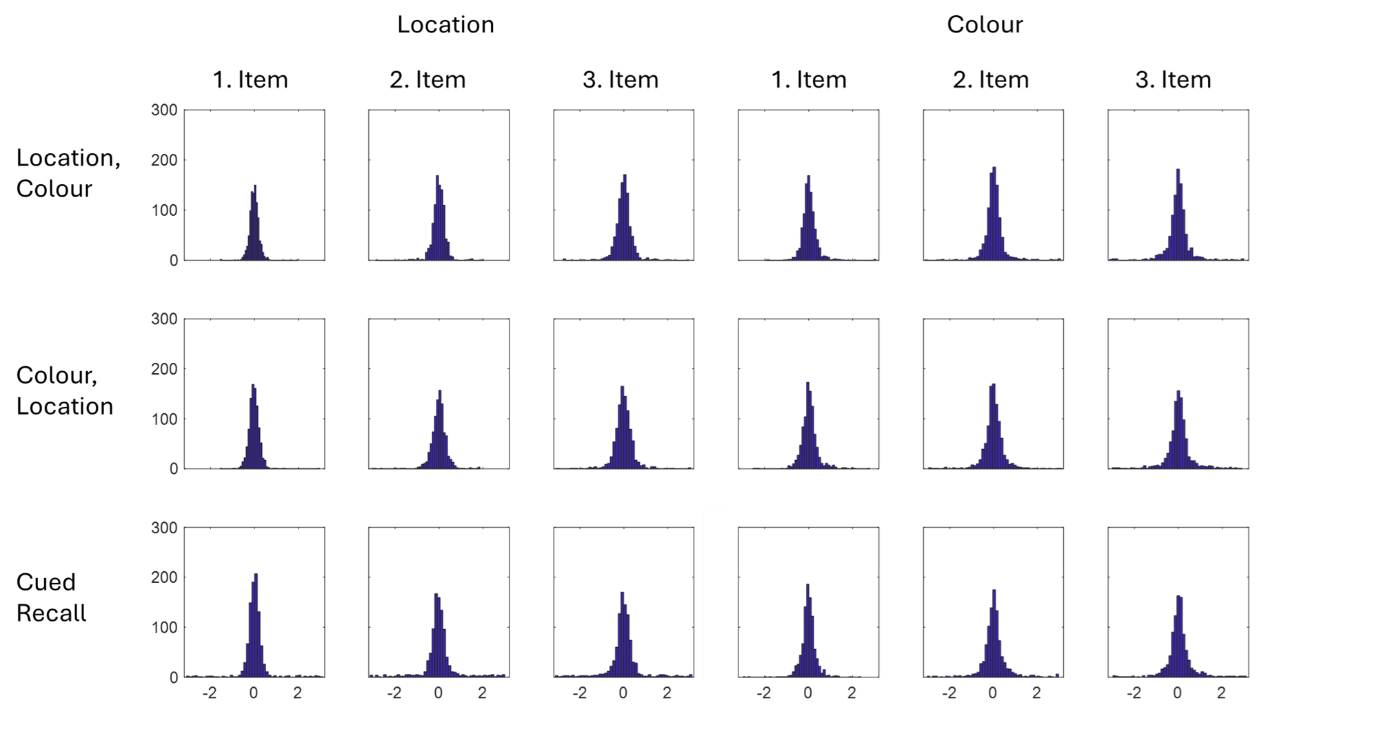


***Order of Reproduction***

The order in which participants reproduced the memory array was investigated for the sequential experiment (**Supplementary Fig. 7**) using a 4x6 repeated-measures ANOVA of the six permutations 1 2 3, 1 3 2, 2 1 3, 2 3 1, 3 1 2 and 3 2 1 and the four probe conditions, whereby the likely order in the uncued conditions was extracted from the full probabilistic model (“Prob Model”). The results show a strong preference for identical presentation and reproduction orders over all conditions.


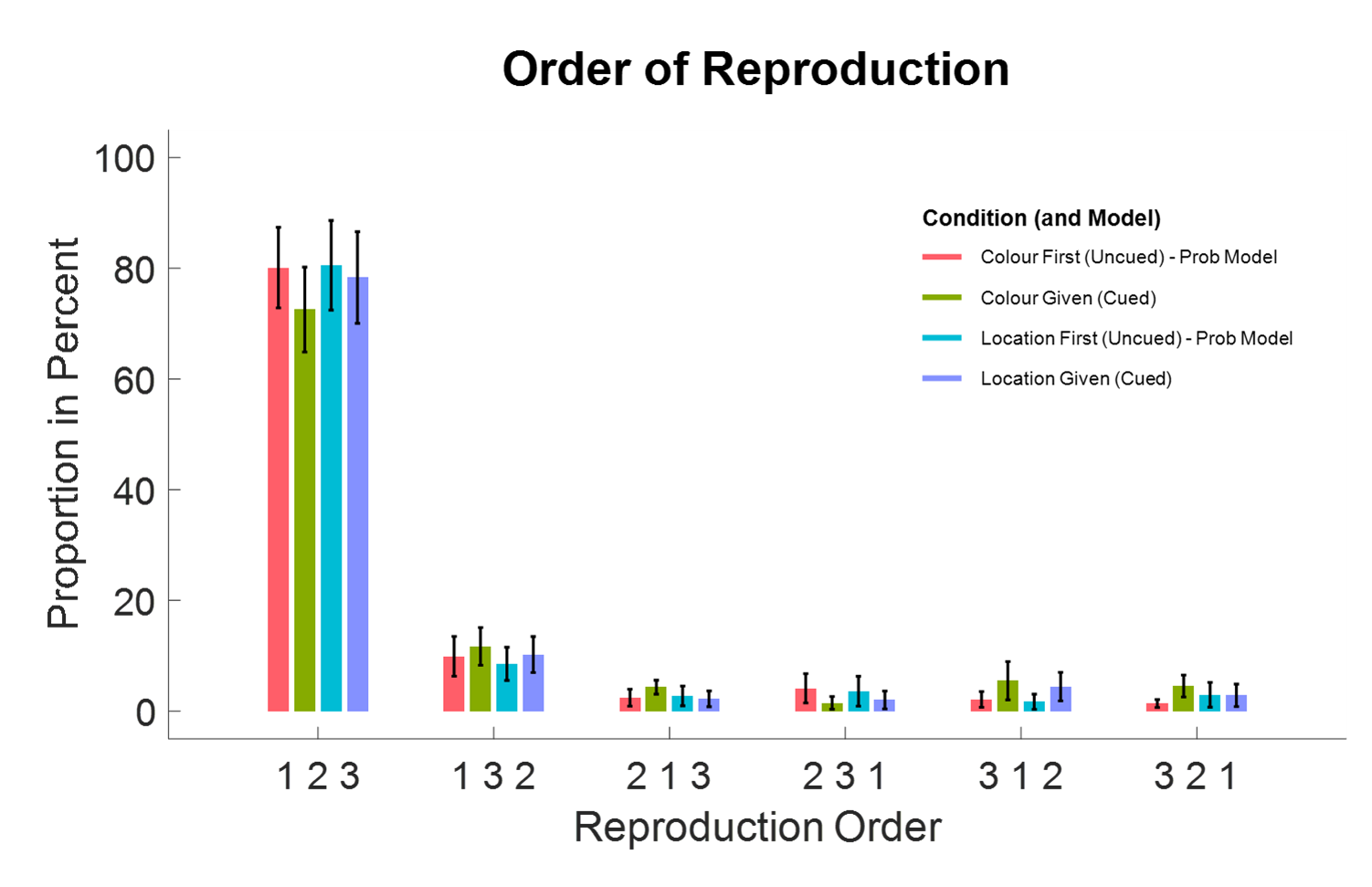


**Supplementary Figure 7 | Order in which participants reproduce the sequential memory array where “1 2 3” is the order in which they have originally seen the stimuli appear on the screen.** In the cued conditions, the order was extracted from the data but in uncued conditions, it was calculated using the full probabilistic model (Prob Model). A clear preference of the order “1 2 3” was consistent over all conditions.

**Supplementary Figure 8 | Bayesian Prior Robustness Analysis for the comparison between Experiment 1 and 2.** The plot illustrates the sensitivity of the Bayes factor BF_10_ to the choice of the prior distribution width for the effect size under the alternative hypothesis H1. The x-axis represents the scale parameter (width) of the Cauchy prior distribution, and the y-axis represents the resulting Bayes factor.


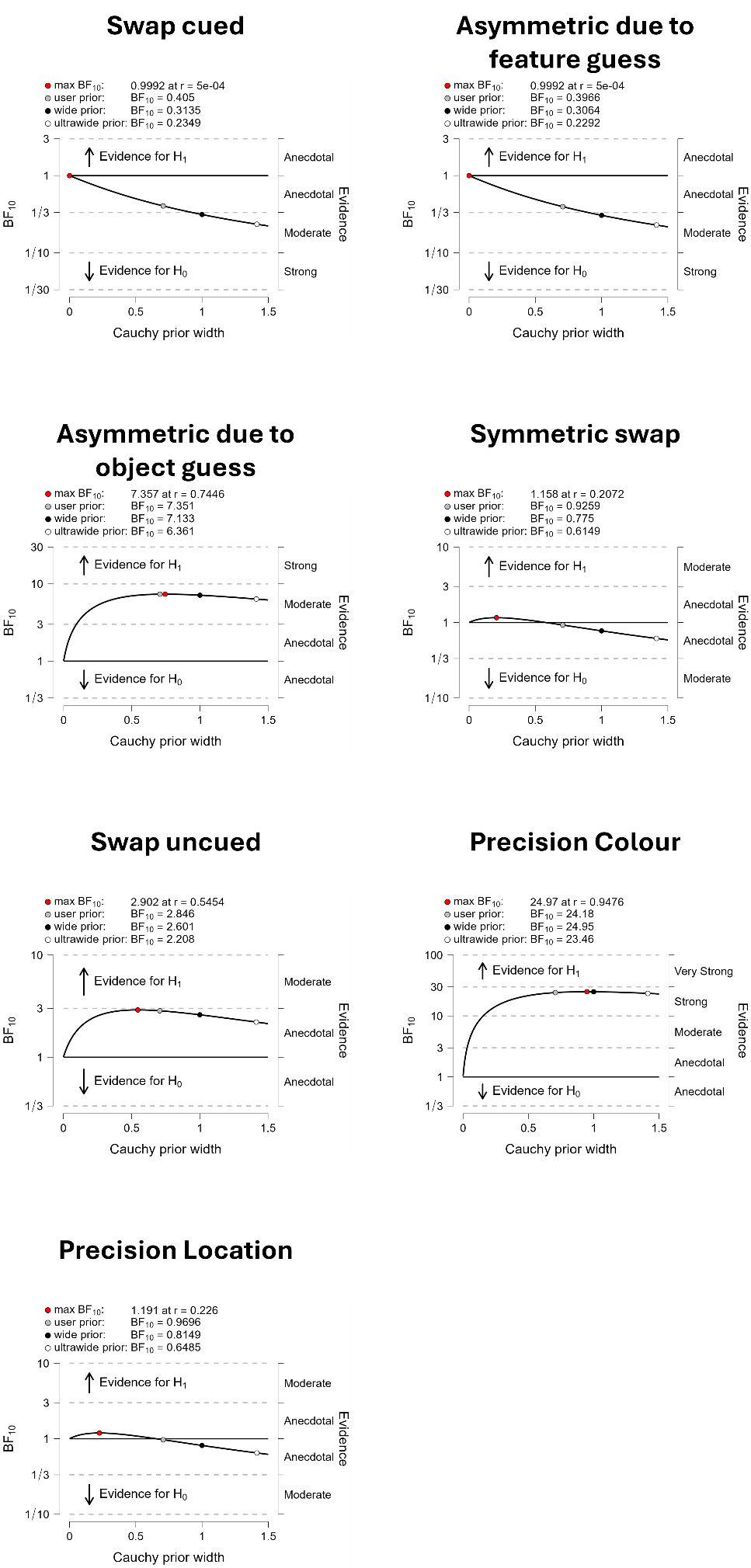


| **Supplementary Table 1: Correlation of the overall “swap” comprising of symmetric misbinding and asymmetric misattribution and the other model parameters** | | | | | |
| --- | --- | --- | --- | --- | --- |
| **Variable** | |  | | **p_swap_mean** | |
| 1. Symmetric_mean |  | Pearson's r |  | -0.0338 |  |
|  |  | BF₁₀ |  | 0.2035 |  |
| 2. prec_col_mean |  | Pearson's r |  | -0.0834 |  |
|  |  | BF₁₀ |  | 0.2255 |  |
| 3. prec_loc_mean |  | Pearson's r |  | -0.1730 |  |
|  |  | BF₁₀ |  | 0.3405 |  |
| 4. Asymmetric_onefeat_mean |  | Pearson's r |  | 0.1009 |  |
|  |  | BF₁₀ |  | 0.2388 |  |
| 5. Asymmetric_twofeat_mean |  | Pearson's r |  | -0.0724 |  |
|  |  | BF₁₀ |  | 0.2187 |  |
| 6. G_sameord_obj_mean |  | Pearson's r |  | 0.6220 |  |
|  |  | BF₁₀ |  | 1089.7323 |  |
| 7. G_sameord_feat_mean |  | Pearson's r |  | 0.2866 |  |
|  |  | BF₁₀ |  | 0.9015 |  |
| 8. G_swap_nonswapped_obj_mean |  | Pearson's r |  | -0.1123 |  |
|  |  | BF₁₀ |  | 0.2494 |  |
| 9. G_swap_nonswapped_feat_mean |  | Pearson's r |  | 0.0734 |  |
|  |  | BF₁₀ |  | 0.2193 |  |
| 10. G_swap_swapped_obj_mean |  | Pearson's r |  | -0.2046 |  |
|  |  | BF₁₀ |  | 0.4233 |  |
| 11. G_swap_swapped_feat_mean |  | Pearson's r |  | 0.0734 |  |
|  |  | BF₁₀ |  | 0.2193 |  |
